# Air, Air Everywhere!

**DOI:** 10.1002/ccr3.3247

**Published:** 2020-08-20

**Authors:** Rafiq Kanji, Kayur Patel, Douglas Stangoe

**Affiliations:** ^1^ East Surrey County Hospital Redhill UK; ^2^ King’s College Hospital London UK

**Keywords:** anesthesia, emergency medicine, intensive care medicine, pneumomediastinum, pneumothorax

## Abstract

When using a gum elastic bougie, a common device to aid difficult intubations, one must appreciate the significant consequences, be prepared to manage emergency complications, and use safely by advancing under direct vision and using the markings to guide insertion.

## Graphical abstract

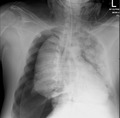

## 1 DESCRIPTION

**Figure 1:**
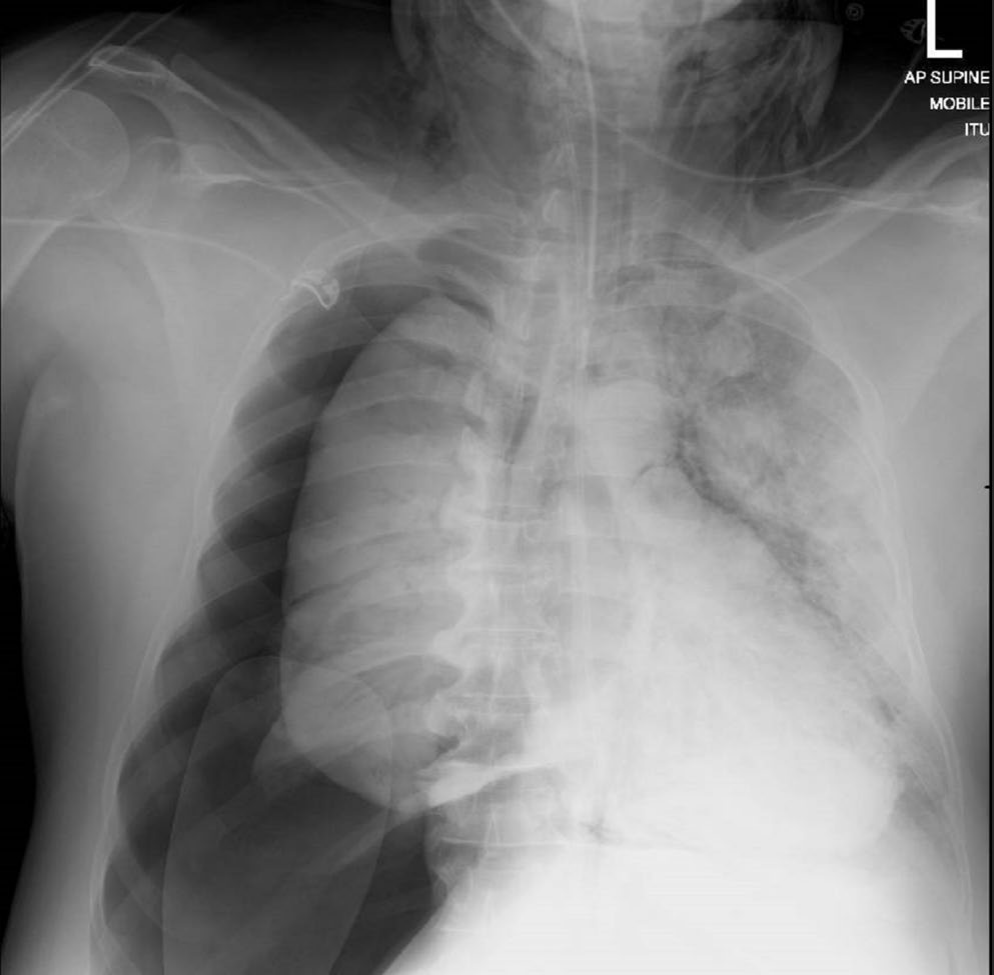
This chest X‐ray shows a large right‐sided pneumothorax with a degree of mediastinal shift suggesting tensioning (The chest X‐ray you should never see!). Furthermore, features of a pneumomediastinum are present including the continuous diaphragm sign, air around the aortic arch, and bilateral subcutaneous emphysema around the neck and the right side of the thorax

A 53‐year‐old African Caribbean male patient tested positive for COVID‐19. He was subsequently intubated and ventilated on the intensive care unit where he developed an endotracheal tube (ETT) cuff leak during his stay. His classical COVID‐19 chest X‐ray showed hazy consolidation and ground‐glass opacification bilaterally. The ETT exchange, done with a gum elastic bougie (GEB), was challenging due to distortion of the glottic inlet by the high volume of thick secretions. As demonstrated in Figure 1, this difficulty caused a significant right‐sided pneumothorax with surgical emphysema secondary to a pneumomediastinum, likely caused by the GEB, or possibly by high airway pressures during invasive ventilation. Additionally, there is a degree of mediastinal shift, though this is difficult to assess due to patient rotation. What is the immediate management? Ideally, an emergency surgical chest drain is inserted concomitantly to prevent exacerbation of the pneumothorax and cause tensioning that may precipitate further hemodynamic instability.^1^ The Cardiothoracic Department's advice was for conservative management with close monitoring and reducing ventilatory pressures, with any further deterioration requiring consideration for a mediastinotomy with drainage of air. The primary teaching point is the significant consequences that can be caused by a GEB, and while a useful instrument, it should be used cautiously and safely.

## CONFLICT OF INTEREST

No author has any conflicts of interest with regard to this case or publication.

## AUTHOR CONTRIBUTIONS

RK: involved in the patient's care during their time on Intensive Care and was able to get the necessary clinical details and images required to submit this report. Additionally, he was involved in writing and assembling the manuscript. KP: involved in the writing the case report and ensuring the clinical image was of an appropriate resolution. DS: involved in writing the case report.
